# MiR-21 Simultaneously Regulates ERK1 Signaling in HSC Activation and Hepatocyte EMT in Hepatic Fibrosis

**DOI:** 10.1371/journal.pone.0108005

**Published:** 2014-10-10

**Authors:** Juan Zhao, Nan Tang, Kaiming Wu, Weiping Dai, Changhong Ye, Jian Shi, Junping Zhang, Beifang Ning, Xin Zeng, Yong Lin

**Affiliations:** 1 Department of Gastroenterology, Shanghai Changzheng Hospital, Second Military Medical University, Shanghai, China; 2 Department of Pharmacology, School of Pharmacy, Second Military Medical University, Shanghai, China; H.Lee Moffitt Cancer Center & Research Institute, United States of America

## Abstract

**Background:**

MicroRNA-21 (miR-21) plays an important role in the pathogenesis and progression of liver fibrosis. Here, we determined the serum and hepatic content of miR-21 in patients with liver cirrhosis and rats with dimethylnitrosamine-induced hepatic cirrhosis and examined the effects of miR-21 on *SPRY2* and *HNF4α* in modulating ERK1 signaling in hepatic stellate cells (HSCs) and epithelial-mesenchymal transition (EMT) of hepatocytes.

**Methods:**

Quantitative RT-PCR was used to determine miR-21 and the expression of *SPRY2, HNF4α* and other genes. Immunoblotting assay was carried out to examine the expression of relevant proteins. Luciferase reporter assay was performed to assess the effects of miR-21 on its predicted target genes *SPRY2* and *HNF4α*. Primary HSCs and hepatocytes were treated with miR-21 mimics/inhibitors or appropriate adenoviral vectors to examine the relation between miR-21 and *SPRY2* or *HNF4α.*

**Results:**

The serum and hepatic content of miR-21 was significantly higher in cirrhotic patients and rats. *SPRY2* and *HNF4α* mRNA levels were markedly lower in the cirrhotic liver. MiR-21 overexpression was associated with enhanced ERK1 signaling and EMT in liver fibrosis. Luciferase assay revealed suppressed *SPRY2* and *HNF4α* expression by miR-21. Ectopic miR-21 stimulated ERK1 signaling in HSCs and induced hepatocyte EMT by targeting *SPRY2* or *HNF4α*. Downregulating miR-21 suppressed ERK1 signaling, inhibited HSC activation, and blocked EMT in TGFβ1-treated hepatocytes.

**Conclusions:**

MiR-21 modulates ERK1 signaling and EMT in liver fibrosis by regulating *SPRY2* and *HNF4α* expression. MiR-21 may serve as a potentially biomarker as well as intervention target for hepatic cirrhosis.

## Introduction

Hepatic fibrosis is characterized by excessive production and deposition of the extracellular matrix (ECM), leading to the destruction of the normal hepatic parenchyma and disruption of the liver structure [1]–[4]. A well-documented event critical to the development of hepatic fibrosis is the activation and proliferation of resident hepatic stellate cells (HSCs) [5]–[7]. Recent evidence also implicates activated fibroblasts in hepatic fibrosis. These activated fibroblasts are transformed from hepatocytes and biliary epithelial cells through the epithelial-mesenchymal transition (EMT) and contribute to liver fibrogenesis [8]–[10].

The extracellular signal-regulated kinase 1 (ERK1) signaling pathway is implicated in both HSC activation and EMT of hepatocytes and biliary epithelial cells. Specifically, activation of the ERK1 signaling pathway promotes HSC activation [11]–[13]. ERK1 is a critical player in this signaling pathway. Our previous study showed that suppression of ERK1 expression could inhibit HSC activation and block EMT of hepatocytes and biliary epithelial cells [14]. Hepatocyte nuclear factor 4α (*HNF4α*), an important transcriptional factor for hepatocyte differentiation and function, is downregulated in human cirrhotic liver [15]. In a rat model of cirrhosis, *HNF4α* significantly suppresses EMT of hepatocytes and alleviates dimethylnitrosamine-induced fibrosis [16]. These findings together indicate that both the ERK1 signaling pathway and EMT may play critical roles in hepatic fibrogenesis and represent a promising therapeutic target in liver fibrosis.

MicroRNAs (miRNAs) are a class of endogenous, small (18–24 nucleotides), non-coding single-stranded RNAs that negatively regulate gene expression through binding to the 3′-untranslated region (UTR) of target mRNAs [17]. Dysregulation of miRNAs contributes to the development of a variety of diseases, including liver fibrosis [18], [19]. MiRNA-21 is highly expressed at the onset of fibrosis in many organs, including the human liver [20]–[22]. Importantly, miR-21 stimulates the proliferation and activation of fibroblasts in different organs with fibrosis, which may involve the PTEN/Akt, NF-kappa B (NF-κB) and ERK1 signaling pathways [20]–[25]. Additional studies further implicate miR-21 in the activation of HSCs [21]. Moreover, our previous study showed that TGFβ1 negatively regulated sprouty2 (*SPRY2*) expression in HSC activation and ectopic *HNF4α* expression in the hepatocytes of rats with fibrotic livers was associated with blocked EMT and reduced miR-21 expression. In this study, a computational algorithm analysis suggested that *SPRY2* and *HNF4α* contain putative miR-21 binding sites. Based on these findings, we speculated that miR-21 could modulate hepatic fibrogenesis by targeting *SPRY2* and *HNF4α* in HSCs and hepatocytes. In the current study, we examined serum and hepatic content of miR-21 in patients with liver cirrhosis and in rats with dimethylnitrosamine-induced hepatic cirrhosis. Effects of miR-21 on *SPRY2* and *HNF4α* in HSCs and hepatocytes were also examined.

## Materials and Methods

### Ethical statements

Written informed consent was obtained from all study participants. Acquisition and use of human tissue specimens or sera were carried out in accordance with established institutional and national ethical guidelines regarding the use of human tissues for research. All experimental procedures were performed in accordance with the Regulations for the Experimental Use of Animals by the State Council of the People's Republic of China. The animals were sacrificed under sodium pentobarbital anesthesia, with efforts to minimize animal suffering in accordance to the ARRIVE guidelines (http://www.nc3rs.org.uk/page.asp?id=1357).

### Computational algorithm analysis

Target sites of miR-21 were predicted using TargetScan (www.targetscan.org/) (Whitehead Institute for Biomedical Research, Cambridge, MA) and PicTar (http://picta.mdc-berlin.de/).

### MiRNAs and adenoviral vectors

MiR-21 mimic, miR-21 inhibitor (anti-miR-21), the control miRNA and small interfering RNAs (siRNAs) against *SPRY2*, *ERK1* and *HNF4α* were synthesized by GenePharma (Shanghai, China). The sequences are listed in Table S1. Replication-deficient E1 and E3 adenoviral vectors, AdERK1, AdSPRY2, AdHNF4α and the control vector-AdGFP that express *ERK1*, *SPRY2*, *HNF4α*, and green fluorescent protein (*GFP*), respectively, as well as adenoviral vectors AdshERK1 (expressing siRNA targeting ERK1 mRNA), AdshHNF4α (expressing siRNA against *HNF4α*) and AdshNC (containing scrambled siRNA) were prepared as previously described [14], [16]. The cDNA fragments of *ERK1*, *SPRY2* and *HNF4α* were generated through RT-PCR from rat HSCs or hepatocytes as detailed elsewhere in the text. The fragments of siRNA against *ERK1* and *HNF4α* (GenePharma) were synthesized, and all of the fragments were inserted into the pAd-Track-Shuttle vector carrying *GFP* gene respectively, to yield pAd-Track vectors (pAd-Track-ERK1, pAd-Track-SPRY2, pAd-Track-HNF4α, pAd-Track-GFP, pAd-Track-shERK1, and pAd-Track-shHNF4α). Homologous recombination was performed using 1 mg *Pme*I-linearized pAd-Track vectors with 0.1 mg supercoiled circular viral backbone vector pAd-Easy-1 in *Escherichia coli* BJ5183 cells. After packaging in 293 cells, recombinant replication-deficient adenoviruses were generated. These adenoviruses could express both of the exogenous genes and *GFP*, allowing direct observation of green fluorescence with microscopy to evaluate the efficiency of infection. None of the adenoviral vectors contained the 3′-UTR region of the related genes.

### Serum and tissue acquisition

Human sera were obtained from 20 healthy individuals and 20 patients with liver cirrhosis (Child-Pugh classification level: B or C). Venous blood samples were collected and stood at room temperature for about 30 min before centrifugation at 820×g for 10 min at 4°C. The supernatant was transferred into new tubes and centrifuged at 16,000×*g* for 10 min at 4°C to completely remove any cell debris. The resulting serum was stored in new Eppendorf tubes at −80°C. Normal or cirrhotic human liver tissues (n = 7 each) were also obtained. The clinical features of the study subjects are shown in Table 1. Additionally, serum and liver tissue specimens were prepared from male Sprague-Dawley (SD) rats (weighing 200–250 g, n = 10) with liver cirrhosis induced by intraperitoneal injection with 1% dimethylnitrosamine (10 µg/kg; Sigma, St. Louis, MO) for 3 consecutive days per week up to 4 weeks [16].

**Table 1:** Clinical features of the patients providing cirrhotic liver tissues.

Clinical characteristics	Number (n = 7)
Age (mean, year)	46.8 (range 39 to 62)
Gender (male/female)	4/3
Etiology	
Hepatitis B	3 (43%)
Hepatitis C	2 (29%)
Alcohol	1 (14%)
Primary biliary cirrhosis	1 (14%)
Child-Pugh classification level (B/C)	4/3

### Cells, transfection and infection

Rat HSC cell line HSC-T6 [26] (kindly provided by Dr. SL. Friedman), which is considered activated mature fibroblast with typical features of myofibroblasts, and human embryonic kidney cell line HEK-293 (ATCC, Manassas, VA) were cultured in Dulbecco's Modified Eagle's Medium (DMEM) (Gibco) with 10% fetal calf serum (FBS) (Gibco). Primary HSCs and hepatocytes were isolated from male SD rats as previously described [16]. Briefly, rats were starved for 18 h and anesthetized with an intraperitoneal injection of 10% chloral hydrate (35 mg/kg). The portal vein was identified and used to perfuse the liver with Krebs Ringer buffer containing EDTA and then with collagenase type IV solution. When the liver showed swelling, lobe bubbling, and the appearance of cellular debris, the liver tissues were bluntly dissected in ice-cold Krebs Ringer Buffer to obtain cell suspension. For HSCs isolation, the cell suspension was repeatedly centrifuged at 50×g (2 min each round) until no visible pellet was observed, followed by filtering through a 250 µm mesh filter to remove tissue fragments. Then, the cell suspension was centrifuged at 200×g for 10 min to yield a pellet of non-parenchymal cells containing the HSCs. At last, HSCs were isolated from the non-parenchymal cell suspension by 8.2% Nycodenz (Nycomed, Oslo, Norway) density gradient centrifugation at 1400×g and extensive washing. The cells were seeded into dishes (35-mm) at 1×10^6^ cells/dish and cultured in DMEM with 10% FBS. For hepatocytes isolation, the cell suspension was filtered through a 250 µm mesh filter, and then through a 60 µm mesh filter to obtain single cell suspension. The suspension was divided into several 50 ml conical tubes, washed and centrifuged at 150×g for 3 min at 4°C for three times. The hepatocytes were seeded into dishes (35-mm) at 1×10^6^ cells/dish and cultured in hepatocyte culture medium as previously described [16]. Trypan blue exclusion showed that cell viability was greater than 90%. For detection of the expression of miRNA and its target genes in activated HSCs and EMT hepatocytes, the cells were cultured for 48 h, followed by stimulating with TGFβ1 (3 ng/ml; 48 h for hepatocytes, 7 days for HSCs). The cell culture media with TGFβ1 was changed every 3 days. In order to determine the effects of upregulating miR-21 on HSC activation and hepatocyte EMT, cells were cultured in medium without TGFβ1 and transfected with miR-21 mimics (100 pmol), or the control miRNA (100 pmol) for 48 h using Lipofectamine™ 2000 (Invitrogen, Carlsbad, CA) as instructed by the manufacturer. To examine the effect of downregulating of miR-21 on HSCs and hepatocytes, the cells were treated with TGFβ1 (3 ng/ml) for 48 h for hepatocytes and 5 days for HSCs. Then, the cells were transfected with miR-21 inhibitors (100 pmol) or its control miRNA (100 pmol) for 48 h. Additionally, cells were infected with appropriate adenoviral vectors at a multiplicity of infection (MOI) of 50 as detailed elsewhere in the text.

### Quantitative real-time reverse transcription polymerase chain reaction (RT-PCR)

Total cellular RNA was extracted from cells, liver tissues or serum using the mirVana RNA isolation kit (Invitrogen). To examine the level of miR-21 in tissues, RNA (2 µg) was polyadenylated with ATP using a poly (A) polymerase (New England BioLabs, Ipswich, MA) at 37°C for 2 h, reverse-transcribed with Super Script III (Invitrogen) and a poly (T) adapter, and subjected to SYBR Green-based real-time PCR analysis (Takara, Dalian, China). To examine the level of miR-21 in serum, we used RT and qPCR kits specifically for accurate miRNA analysis (Applied Biosystems). A TaqMan microRNA Reverse Transcription Kit (Applied Biosystems, USA) was used to perform RT reactions. Then, the RT reactions were incubated for 30 min at 16°C, 30 min at 42°C, 5 min at 85°C, followed by maintaining at 4°C. For real-time PCR, 1.33 µl diluted RT products were mixed with 10 µl of 2× Taqman PCR master mixture (No AmpErase UNG), 1 µl TaqMan MicroRNA Assay and Nuclease-free water in a final volume of 20 µl. All reactions were run on the ABI 7300 (Applied Biosystems, USA) with the following conditions: 95°C for 30 s followed by 40 cycles at 95°C for 10 s, and 60°C for 30 s. For detection of mRNA transcript levels, total RNA was reverse-transcribed using Super Script III with random primers and subjected to SYBR Green-based real-time PCR analysis. MiR-21 was normalized against miR-238 in the serum and against *Rnu6-2* in liver tissues. The mRNA was normalized against *β-actin*. All primer sequences are listed in Table S2.

### Western blotting assays

Proteins were extracted with RIPA lysis buffer (Bioteke Co, Beijing, China) containing a cocktail of protease inhibitors and phosphatase inhibitors, and separated by sodium dodecyl sulfate (SDS)-polyacrylamide gel electrophoresis and transferred to a nitrocellulose membrane (HAHY00010, Millipore, Billerica, MA). The membrane was probed with specific primary antibodies (Table S3) overnight at 4°C, followed by incubation with appropriate donkey-anti-mouse or donkey-anti-rabbit secondary antibodies (GIBCO BRL, Rockville, CA). Protein bands were visualized using the Odyssey infrared imaging system (LI-COR Biotechnology, NE). GADPH was used as a loading control.

### Cell migration assays

The migration of HSC-T6 cells was examined using a transwell assay [27]. Briefly, 1×10^4^ cells transfected with miR-21 inhibitors or the control miRNA were seeded in transwell inserts with 8.0- µm-pore transwell filters (BD, NJ), and the wells under the inserts were coated with fibronectin (50 µg/ml) and 500 µl DMEM containing 10% FBS was added as chemoattractants. After 24 and 48 h, the migration of HSCs was quantified by counting the number of cells stained by 0.5% crystal violet.

### Luciferase reporter assay

Reporter plasmids psiCHECK-2-SPRY2 and psiCHECK-2-HNF4α were constructed as described previously [28]. The 280-bp fragment of the *SPRY2* 3′-UTR containing the miR-21 target sequence was amplified from total DNA of primary rat HSCs by PCR and cloned into the psiCHECK-2 dual luciferase reporter plasmid (Promega, Madison, WI) at the 3′-end of the coding sequence of *Renilla reniformis* luciferase to produce psiCHECK-2-SPRY2. Similarly, the luciferase reporter plasmid psiCHECK-2-HNF4α containing a 280-bp fragment of the *HNF4α* 3′-UTR with the miR-21 target sequence was prepared. To determine sequence specificity, we also constructed the plasmids psiCHECK-2-mt-SPRY2 or psiCHECK-2-mt-HNF4α in which the conserved targeting sequence of miR-21 was mutated (from AUAAGCU to CUCGAGC). MiR-21 mimics and reporter plasmids were co-transfected into HEK-293 cells using Lipofectamine™ 2000. Renilla constructs were transfected as an internal control. After 48 h incubation, firefly luciferase and Renilla luciferase activities were measured using a luciferase assay system (Promega). All luciferase activity readings were normalized relative to the activity of the Renilla luciferase control. All experiments were performed in triplicate.

### Statistical analysis

Analysis of variance (ANOVA) and Student's *t*-test were used for comparison of normally distributed data between the groups and paired data. Data not normally distributed were compared using the Mann-Whitney test. All results were reported as mean ± SD of at least three independent experiments and a *P* value <0.05 was considered statistically significant.

## Results

### Liver fibrosis is associated with broad changes in the expression of miR-21 and its targeted genes, ERK1 signaling and EMT-associated genes

**Figure 1:**
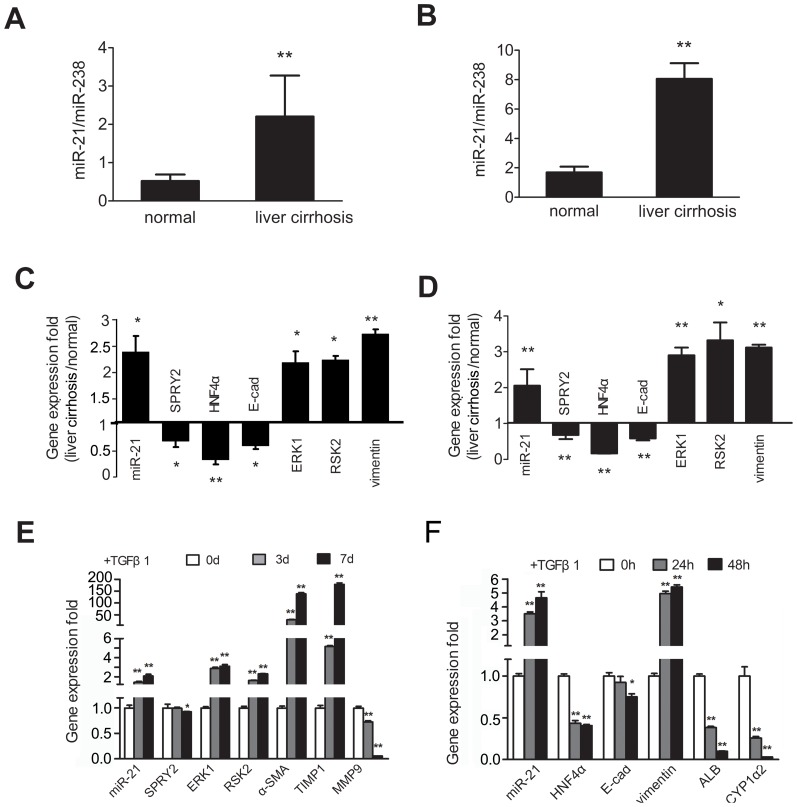
Liver fibrosis is associated with broad changes in the expression of miR-21 and its targeted genes, ERK1 signaling and EMT-associated genes. Serum miR-21 contents in cirrhotic patients (n = 20) and normal subjects (n = 20) (A) and in rats with dimethylnitrosamine-induced liver cirrhosis (B). MiR-21 expression was normalized against miR-238 in serum. **P<0.01 vs. normal controls. Quantitative RT-PCR examination of the expression of miR-21 and its targeted genes *SPRY2* and *HNF4α*, *ERK1* and its downstream target *RSK2*, and epithelial-mesenchymal transition (EMT)-associated genes *E-cadherin* and *vimentin* in the liver tissues of cirrhotic patients (n = 7) (C) and rats (n = 10) (D). *P<0.05 and **P<0.01 vs. normal subjects or rats. Primary HSCs were treated with TGFβ1 for 7 days (E) and primary hepatocytes for 48 h (F) as detailed in methods. Quantitative RT-PCR examination of the expression of miR-21 and its targeted genes *SPRY2* and *HNF4α, ERK1* and *RSK2*, *E-cadherin*, *vimentin* and *MMP-9*, liver fibrogenesis-associated genes α*-SMA* and *TIMP1*, and liver specific genes *ALB* and *CYP1a2*. MiR-21 expression was normalized against *Rnu6-2* and mRNA expression was normalized against *β-actin*. *P<0.05 and **P<0.01 vs. non-stimulated HSCs or hepatocytes. Each value represents the mean with the SD (error bars) for triplicate samples.

We first examined the expression of miR-21 in the serum of patients with hepatic cirrhosis or rats with dimethylnitrosamine-induced liver cirrhosis, which was confirmed by histopathologic examination (Fig. S1A and S1B). The serum content of miR-21 was 2.3 fold higher in patients with liver cirrhosis than in healthy control subjects (Fig. 1A). Significantly higher serum miR-21 content was also seen in the cirrhotic rats (Fig. 1B). Consistently, we also observed a marked increase in the miR-21 content in the cirrhotic liver tissues of humans and rats (Fig. 1C and 1D). Our quantitative RT-PCR further revealed that the mRNA transcript levels of *ERK1* and its downstream target ribosomal protein S6 kinase 2 (*RSK2*) were significantly higher in the cirrhotic liver tissues (Fig. 1C and 1D). The mRNA transcript levels of EMT-associated gene *vimentin* were approximately 2.6 to 3.1 fold higher in the cirrhotic liver of both humans and rats (Fig. 1C and 1D) while those of *E-cadherin* were markedly lower compared to normal livers (Fig. 1C and 1D). Screening for targets of miR-21 using Target Scan and PicTar yielded *SPRY2* and *HNF4α* as the putative targets. Our RT-PCR assays further showed that the mRNA transcript levels of both *SPRY2* and *HNF4α* were markedly lower in the cirrhotic liver of both humans and rats (Fig. 1C and 1D).

Additionally, TGFβ1 markedly increased miR-21 levels in primary HSCs and hepatocytes of rats in a time-dependent manner (Fig. 1E and 1F). Similar increase was observed in the transcript levels of *ERK1* and *RSK2* and fibrogenesis-associated genes α-smooth muscle actin (α*-SMA*) and tissue inhibitor of metalloproteinase 1 (*TIMP1*) in primary HSCs (Fig. 1E). The mRNA transcript levels of *SPRY2* in primary HSCs (Fig. 1E) and *HNF4α* in primary hepatocytes (Fig. 1F) were noticeably decreased. Meanwhile, the mRNA transcript levels of *vimentin* were markedly higher while those of *E-cadherin* were markedly lower in the primary hepatocytes. Furthermore, TGFβ1 caused a marked reduction in certain liver specific genes, including albumin (*ALB*) and cytochrome P450 (CYP) 1a2 (*CYP1a2*) in primary hepatocytes (Fig. 1F). Western blotting assays additionally showed that *SPRY2* and *HNF4α* expression was markedly suppressed in TGFβ1-treated HSCs or hepatocytes (Fig. S2A and S2C) and cirrhotic rat liver tissues (Fig. S2B and S2D).

### MiR-21 targets the 3′-UTR of SPRY2 and HNF4α

**Figure 2:**
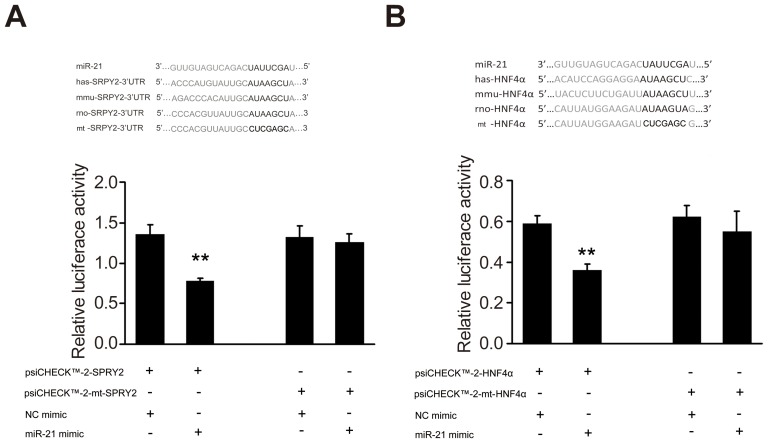
MiR-21 directly targets the 3′-UTR of *SPRY2* and *HNF4α*. The sequences of putative miR-21 binding sites in the 3′-UTR of *SPRY2* and *HNF4α* are shown (upper panels, A and B) with the mutated sequences CUCGAGC. HEK-293 cells were transfected with miR-21 mimics and *SPRY2* (A) or *HNF4α* 3′-UTR reporter plasmids (B) containing putative wild type miR-21 binding sites or mutant miR-21 binding sites. Luciferase activities were examined and normalized against Renilla constructs. **P<0.01 vs. controls. Each value represents the mean with the SD (error bars) for triplicate samples of at least three independent experiments.

As our computational algorithm analysis showed that *SPRY2* and *HNF4α* were candidate target genes of miR-21, we investigated whether miR-21 directly targeted *SPRY2* and *HNF4α* expression by co-transfecting *SPRY2* or *HNF4α* 3′-UTR reporter plasmids containing putative miR-21 binding sites and miR-21 mimics into HEK-293 cells. Luciferase assays showed that miR-21 mimics caused a 42.6% and 41.4% reduction in luciferase activities of cells transfected with *SPRY2* 3′-UTR (Fig. 2A) and *HNF4α* 3′-UTR reporter plasmids(Fig. 2B), respectively. Co-transfection of miR-21 mimics and *SPRY2* or *HNF4α* 3′-UTR reporter plasmids containing mutant miR-21 binding sites, on the other hand, failed to suppress luciferase activities (Fig. 2A and 2B). These results indicated that miR-21 directly targeted *SPRY2* and *HNF4α* mRNA to modulate their expression.

### MiR-21 regulates ERK1 signaling in HSCs by targeting SPRY2

**Figure 3:**
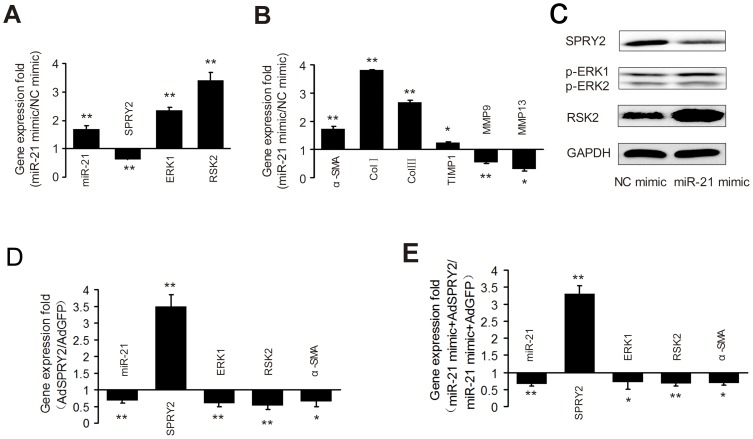
MiR-21 and *SPRY2* mutually modulate the expression of each other in regulating ERK1 signaling in HSCs. Primary HSCs were treated with miR-21 mimics for 48 h, MiR-21 and the mRNA transcript levels of *SPRY2*, *ERK1* and *RSK2* (A), *MMP-9* and *MMP-13*, fibrogenesis-associated genes α*-SMA*, *TIMP1*, *Col I* and *Col III* (B) were examined by quantitative RT-PCR. Immunoblotting assays were done to detect the expression of *SPRY2*, *phospho-ERK1*, *phospho-ERK2* and *RSK2* (C). GADPH was used as a loading control. Blots representative of at least three independent experiments are shown. Primary HSCs treated with TGFβ1 for 48 h were infected by AdSPRY2 (D) and primary quiescent HSCs were transfected with miR-21 mimics followed by AdSPRY2 infection 48 h later (E). Then, the mRNA transcript levels of *SPRY2*, *ERK1*, *RSK2* and *α-SMA* were examined by quantitative RT-PCR. MiR-21 expression was normalized against *Rnu6-2* and mRNA expression was normalized against *β-actin*. Each value in (A), (B), (D) and (E) represents the mean with the SD (error bars) for triplicate samples of at least three independent experiments. *P<0.05 or **P<0.01 vs. controls.

We further examined the effects of miR-21 and *SPRY2* on each other and on ERK1 signaling in HSCs. We found that miR-21 mimics caused a marked reduction in the mRNA transcript and protein levels of *SPRY2* in primary quiescent HSCs (Fig. 3A–3C). Meanwhile, we observed that miR-21 mimics caused a noticeable increase in the mRNA transcript and protein levels of *ERK1* and *RSK2*. In addition, AdSPRY2 infection of primary HSCs treated with TGFβ1 for 48 h suppressed the expression of miR-21 accompanied by a marked decline in the mRNA transcript levels of *ERK1* and *RSK2* (Fig. 3D), whereas miR-21 level slightly increased after primary HSCs were transfected with siRNA against *SPRY2* (Fig. S3). These results suggest the presence of a modulatory loop between miR-21 and *SPRY2*. We then transfected primary quiescent HSCs with miR-21 mimics followed by AdSPRY2 infection 48 h later. We found that *SPRY2* significantly attenuated miR-21-induced increase in the mRNA transcript levels of *ERK1* and *RSK2* (Fig. 3E), suggesting that *SPRY2* may function downstream of miR-21. Moreover, AdERK1 is synergetic with miR-21 mimics for the activation of ERK1 signaling in primary HSCs, and AdshERK1 could enhance the inhibitory effect of miR-21 inhibitor on the ERK1 signaling pathway in activated HSCs (Fig.S4A and S4B).

### MiR-21 promotes EMT of primary hepatocytes by targeting HNF4α

**Figure 4:**
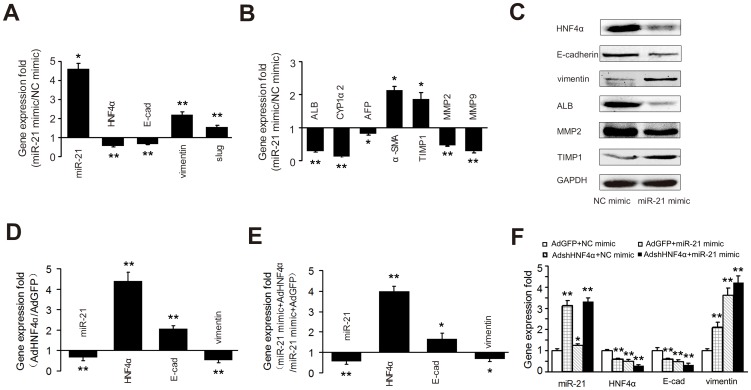
MiR-21 and *HNF4α* mutually modulate the expression of each other in regulating EMT of primary hepatocytes. Primary rat hepatocytes were treated with miR-21 mimics for 48 h. MiR-21 and the mRNA transcript levels of *HNF4α*, EMT associated genes *E-cadherin*, *vimentin* and *slug* (A), *MMP-2* and *MMP-9*, fibrogenesis-associated genes α*-SMA* and *TIMP1* and liver specific genes *ALB*, *CYP1a2* and *AFP* (B) were examined by quantitative RT-PCR. *HNF4α*, *E-cadherin* and *vimentin*, ALB, *MMP-2* and *TIMP1* were examined by Western blotting assays (C). GADPH was used as a loading control. Blots representative of at least three independent experiments are shown. Primary hepatocytes were treated with TGFβ1 for 48 h followed by AdHNF4α infection 48 h later (D). Primary hepatocytes were transfected with miR-21 mimics followed by AdHNF4α (E) or AdshHNF4α (F) infection 48 h later. MiR-21 and the mRNA transcript levels of *HNF4α*, *E-cadherin* and *vimentin* were then examined by quantitative RT-PCR. Each value in (A), (B), (D), (E) and (F) represents the mean with the SD (error bars) for triplicate samples of at least three independent experiments and miR-21 expression was normalized against *Rnu6-2* and mRNA expression was normalized against *β-actin*. *P<0.05 or **P<0.01 vs. controls.

Given our current finding that miR-21 directly targeted *HNF4α* and our previous finding that *HNF4α* significantly suppressed the EMT of hepatocytes [16], we further examined the effects of miR-21 and *HNF4α* on each other and on the EMT of primary hepatocytes. As shown in Fig. 4A, 4B and 4C, miR-21 mimics caused a marked reduction in the mRNA and protein levels of *HNF4α* in primary isolated hepatocytes along with a noticeable increase in the mRNA and protein levels of *vimentin* and a significant decrease in the mRNA transcript levels of *E-cadherin*. Similar results were observed in TGFβ1-treated hepatocytes (Fig. S5). AdHNF4α infection of primary hepatocytes that had been treated by TGFβ1 for 48 h decreased miR-21 levels, along with a significant reduction in the mRNA transcript levels of *vimentin* and a noticeable increase in the mRNA transcript levels of *E-cadherin* (Fig. 4D). We then transfected primary hepatocytes with miR-21 mimics followed by AdHNF4α infection 48 h later. We found that *HNF4α* significantly suppressed miR-21 and the mRNA transcript levels of *vimentin* while it markedly increased the mRNA transcript levels of *E-cadherin* (Fig. 4E), indicating that *HNF4α* may function downstream of miR-21. Furthermore, we transfected primary hepatocytes with miR-21 mimics followed by AdshHNF4α infection 48 h later. We found that AdshHNF4α slightly enhanced miR-21 level in primary hepatocytes (Fig. 4F). AdshHNF4α and miR-21 alone or in combination reduced the mRNA transcript levels of *E-cadherin*, but increased the mRNA-transcript levels of *vimentin*. These results suggest the presence of a modulatory loop between miR-21 and *HNF4α*.

### Downregulation of miR-21 targets ERK1 signaling and SPRY2 in HSC activation and blocks EMT of TGFβ1-treated hepatocytes by targeting HNF4α

**Figure 5:**
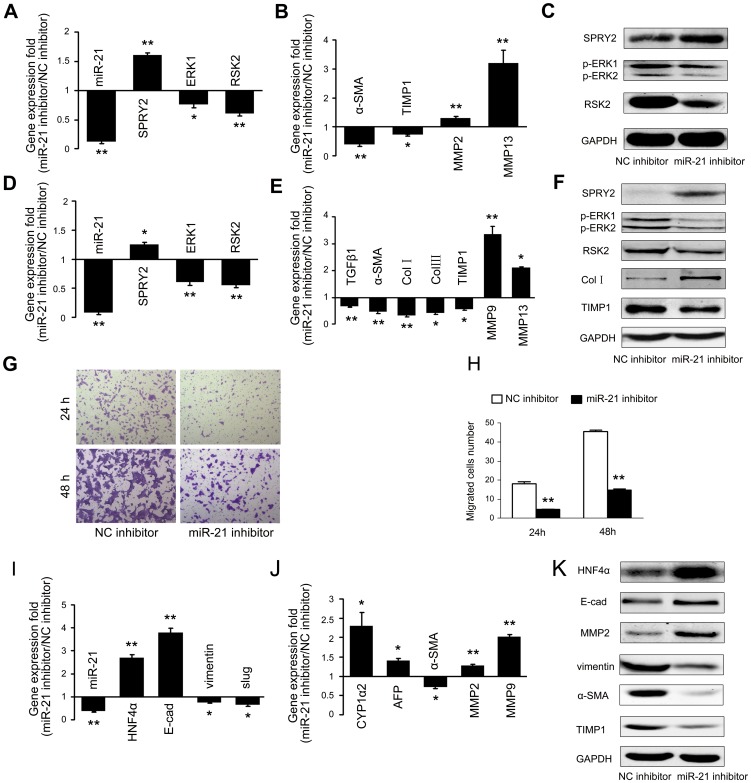
MiR-21 inhibitors target ERK1 signaling and *SPRY2* in HSC activation and block EMT of TGFβ1-treated hepatocytes by targeting *HNF4α*. Primary HSCs were treated with TGFβ1 for 5 d followed by transfection with miR-21 inhibitors. MiR-21 and the mRNA transcript levels of *SRPY2*, *ERK1* and *RSK2* (A), *α-SMA*, *TIMP1*, *MMP-2* and *MMP-13* (B) were examined by quantitative RT-PCR. Immunoblotting assays were done to detect the expression of *SPRY2*, *phospho-ERK1*, *phospho-ERK2* and *RSK2* (C). HSC-T6 cells were transfected with miR-21 inhibitors. MiR-21 and the mRNA transcript levels of *SRPY2*, *ERK1* and *RSK2*, *MMP-9*, *MMP-13*, fibrogenesis-associated genes *TGFβ1*, α*-SMA*, *Col I*, *Col III* and *TIMP1* were examined by quantitative RT-PCR (D, E). Immunoblotting assays were done to detect the expression of *SPRY2*, *phospho-ERK1*, *phospho-ERK2* and *RSK2*, *Col I* and *TIMP1* (F). HSC-T6 cells were transfected with miR-21 inhibitors and transwell cell migration assays were performed as described in methods (G, H). Hepatocytes were treated with TGFβ1 for 48 h followed by transfection with miR-21 inhibitors. MiR-21 and the mRNA transcript levels of *HNF4α*, *E-cadherin* and *vimentin*, *MMP-2*, *MMP-9* and α*-SMA*, liver specific genes *CYP1a2* and *AFP* were examined by quantitative RT-PCR (I, J). Immunoblotting assays were done to detect the expression of *HNF4α*, *E-cadherin* and *vimentin*, *MMP2*, *α-SMA* and *TIMP1* (K). GADPH was used as a loading control. Blots representative of at least three independent experiments are shown. MiR-21 expression was normalized against *Rnu6-2* and mRNA expression was normalized against *β-actin*. Each value in (A), (B), (D), (E), (H), (I) and (J) represents the mean with the SD (error bars) for triplicate samples of at least three independent experiments. *P<0.05 or **P<0.01 vs. controls.

To determine whether downregulating miR-21 impacted on ERK1 signaling and HSC activation, we transfected TGFβ1-treated primary HSCs and HSC-T6 cells with miR-21 inhibitors and found that miR-21 inhibitors markedly reduced the levels of miR-21 along with an apparent decline in the mRNA transcript levels of *ERK1* and its downstream target *RSK2* (Fig. 5A and 5D). Furthermore, miR-21 inhibitors significantly upregulated the mRNA transcript levels of *SPRY2*. These findings were further confirmed by immunoblotting assays (Fig. 5C and 5F). Expectedly, miR-21 inhibitors markedly reduced the expression of profibrotic genes in activated HSCs, including *TGFβ1*, *α-SMA*, *Col I* and *III*, and *TIMP1*, and augmented the expression of *MMP-9* and *MMP-1*3 (Fig. 5B, E and 5F). The migration ability of HSC-T6 cells was also significantly suppressed by miR-21 downregulation (Fig. 5G and 5H). The results showed that downregulation of miR-21 could inhibit ERK1 signaling and HSC activation by targeting *SPRY2*. Moreover, miR-21 inhibitors also caused a significant increase in the mRNA and protein levels of *HNF4α*, along with enhanced mRNA transcript levels of *E-cadherin* and certain liver specific genes, and attenuated the expression of a cluster of mesenchymal markers and profibrotic genes in TGFβ1-treated hepatocytes (Fig. 5I-5K). The data revealed that decreased miR-21 expression could block the TGFβ1-induced EMT process in hepatocytes by directly promoting *HNF4α* expression.

## Discussion

It is widely accepted that ERK1 signaling plays a vital role in HSC activation in hepatic fibrogenesis [13], [14]. Additionally, hepatocyte undergoing EMT contributes to the accumulation of fibrogenic myofibroblasts [10], [16]. Blocking EMT may hold the promise of reversing liver fibrogenesis. Our previous study demonstrated that targeted co-suppression of ERK1 signaling in HSCs and EMT of hepatocytes could inhibit transformation of liver parenchymal and mesenchymal cells to activated fibroblasts, and significantly attenuate hepatic fibrosis [14], [16]. These results prompted us to speculate that the therapeutic strategy targeting the ERK1 signaling pathway in HSCs and EMT of hepatocytes may render the fibrotic liver more amenable to treatment compared with conventional therapy.

Aberrant expression of miRNAs has recently been documented in hepatic fibrogenesis, including miR-21, 122, -29, -19, -200, and -34 [29]–[34]. Prominent among these dysregulated miRNAs is miR-21, which has been shown to promote fibrogenic activation of fibroblasts [20], [35] and to be implicated in the amplification of a series of important cellular signaling pathways [21], [36], [37]. However, up to date, whether miR-21 could regulate multiple signaling pathways simultaneously in hepatic fibrogenesis is still unclear.

In the current study, we observed markedly increased miR-21 contents in the serum and hepatic tissues of patients with liver cirrhosis and rats with dimethylnitrosamine-induced hepatic cirrhosis. The findings are consistent with the report of upregulated miR-21 in fibrotic liver biopsies of HCV patients [22]. Recently, certain dysregulated miRNAs have been considered as non-invasive candidate diagnostic biomarkers for liver diseases [38], [39]. It may be worthwhile to investigate whether plasma miR-21 could serve as a diagnostic marker or prognostic predictor in cirrhotic patients.

ERK1 signaling and EMT are known to have significant impact on fibrogenesis[14], [16], [40]. *Spry1-4* is potent inhibitor of the MAPK pathway and *SPRY2* is abundant in the liver [41]–[43]. Previous studies have shown that miR-21 contributes to colon cancer and gliomas along with downregulation of *SPRY2* to stimulate ERK signaling [44], [45]. Additionally, *HNF4α*, the liver-enriched transcription factor, could attenuate hepatic fibrosis by improving liver function and alleviating EMT in hepatic fibrosis [15], [16]. Interestingly, our computational prediction suggests that miR-21 may directly target the 3′-UTR of *SPRY2* and *HNF4α*. Our luciferase reporter assays further demonstrated that miR-21 downregulated *SPRY2* and *HNF4α* expression. We also found that overexpression of *SPRY2* and *HNF4α* led to significantly subdued levels of miR-21 in HSCs and hepatocytes, suggesting the presence of autoregulatory feedback loops between miR-21 and *SPRY2* or *HNF4α*.

To further confirm that dysregulated miR-21 simultaneously promotes fibrotic transformation of HSCs and hepatocytes, we treated primary HSCs or hepatocytes with miR-21 mimics. Expectedly, our data suggested that miR-21 could contribute to the accumulation of fibroblasts not only by stimulating HSC activation via inhibiting *SPRY2* expression to increase ERK1 signaling, but also by triggering EMT of hepatocytes via downregulating *HNF4α*. In view of previous studies and our present work, miR-21 is integrally involved in multiple pathways and profibrotic network in fibroblast transformation of quiescent HSCs and hepatocytes, including TGFβ1/smad, NF-κB, ERK1 signaling and EMT, suggesting that miR-21 may serve as a ‘super’ regulatory miRNA in liver fibrosis. To our knowledge, this is the first report of the roles of miR-21 in simultaneous myofibrobalst transformation of both liver parenchymal and mesenchymal cells in liver fibrosis.

Interestedly, our results showed miR-21 and *SPRY2*/*HNF4α* mutually modulated the expression of each other, suggesting that there was a regulatory feedback loop between miR-21 and its target genes. The precise mechanism, however, remains unclear. It has been documented that some target genes could bind to the regulatory miRNA to influence the level of the miRNA. For example, our previous study has found that LIN28A, the target gene of miR-370, blocked the biogenesis of miR-370 by binding to its precursor [46]. We speculate that *SPRY2* and *HNF4α* might exert the feedback regulatory effect with the similar mechanism.

The enhanced effect of miR-21 on EMT and ERK1 singling prompted us to propose that inhibiting miR-21could ameliorate biological characteristics of activated HSCs as well as block EMT of TGFβ1-treated hepatocytes. As expected, inhibiting miR-21 upregulated *SPRY2* and restrained ERK1 signaling in activated HSCs, and subsequently suppressed the migration of activated HSCs. Moreover, we demonstrated that decreased miR-21 expression in hepatocytes undergoing EMT could increase levels of *HNF4α* and epithelial markers, restore expression of certain liver specific genes in hepatocytes. The above data further showed that downregulated miR-21 expression exerted the inhibitory effects on ERK1 signaling in HSCs and EMT of hepatocytes simultaneously during hepatic fibrosis.

Taken together, our results revealed significant elevation of circulating, hepatic and cellular miR-21 expression, which is associated with ERK1 signaling and EMT in liver fibrosis. MiR-21 directly interacts with the 3′-UTR of *SPRY2* and *HNF4α*, leading to enhanced ERK1 signaling in HSCs and hepatocyte EMT. Downregulating miR-21 suppressed ERK1 signaling, inhibited HSC activation, and blocked EMT in TGFβ1-treated hepatocytes. Our data strongly indicated the presence of broad changes in miR-21 expression and its targeted genes, ERK1 signaling and EMT-associated genes in hepatic fibrosis and that miR-21 may be a central player in hepatic fibrogenesis. MiR-21 could serve as a potentially clinically useful biomarker and represent a promising molecular target for hepatic fibrosis.

## Supporting Information

Figure S1 **Histopathologic examination of the liver tissue of cirrhotic patients and rats.** Histopathologic examination of the liver tissue from the patient with hepatic cirrhosis (A). H&E staining of the liver tissue from rats with dimethylnitrosamine-induced liver cirrhosis. The presence of cirrhosis is further shown by Van Gieson (VG), Masson and Sirius Red staining (B).
(TIF)

Figure S2 **Protein levels of** ***SPRY2*** **and** ***HNF4α*** **in TGFβ1 treated cells and cirrhotic rat liver tissues.** The levels of *SPRY2* and *HNF4α* were examined by Western blotting assays in primary HSCs treated with TGFβ1 for 7 days (A) and primary hepatocytes treated with TGFβ1 for 48 h (C). Representative images of levels of *SPRY2* and *HNF4α* in cirrhotic rat liver tissue are shown (B and D). The proteins were extracted from normal (n = 3) and cirrhotic liver tissues (n = 3) randomly. The figures only showed the representative images. GAPDH was used as a loading control.
(TIF)

Figure S3 **Effect of siRNA against** ***SPRY2*** **on miR-21 level.** Primary rat HSCs were transfected with siRNA against *SPRY2*. Cells were collected 48 h after siRNA delivery. Quantitative RT-PCR was carried out to detect miR-21 expression. Gene expression folds were normalized against the control. Each value represents the mean with the SD for triplicate samples. (*P<0.05).
(TIF)

Figure S4 **Associative action of** ***ERK1*** **and miR-21.** Primary rat HSCs were treated with AdERK1 and miR-21 mimic (A) or primary HSCs were treated with TGFβ1 for 48 h followed by AdshERK1 and miR-21 inhibitor transfection for 48 h (B). Quantitative RT-PCR was carried out to detect the levels of *miR-21*, *ERK1*, *RSK2* and *α-SMA*. Gene expression folds were normalized against the control. Each value represents the mean with the SD for triplicate samples. (*P<0.05, **P<0.01).
(TIF)

Figure S5 **Combined effect of miR-21 mimics and TGFβ1 on EMT in primary rat hepatocytes.** Primary rat hepatocytes were treated with miR-21 mimics and TGFβ1 for 48 h. MiR-21 level was examined by quantitative RT-PCR (A). Expression of *E*-cadherin and *vimentin* was examined by Western blotting assays (B). GADPH was used as a loading control. Blots representative of at least three independent experiments are shown.
(TIF)

Table S1 **Sequences for miRNA mimic and inhibitor, siRNA and 3′-UTR.**
(DOC)

Table S2 **Primer sequences for real-time polymerase chain reaction.**
(DOC)

Table S3 **Primary antibodies used for western blotting analysis.**
(DOC)

## References

[1] Friedman SL (2008) Mechanisms of hepatic fibrogenesis. Gastroenterology 134: 1655–1669 10.1053/j.gastro.2008.03.003. PubMed: 18471545 18471545 PMC2888539

[2] Castera L (2012) Noninvasive methods to assess liver disease in patients with hepatitis B or C. Gastroenterology 142: 1293–1302 10.1053/j.gastro.2012.02.017. PubMed: 22537436 22537436

[3] Starr SP, Raines D (2011) Cirrhosis: diagnosis, management, and prevention. Am Fam Physician 84: 1353–1359. PubMed: 22230269. 22230269

[4] Hernandez-Gea V, Friedman SL (2011) Pathogenesis of liver fibrosis. Annu Rev Pathol 6: 425–456 10.1146/annurev-pathol-011110-130246. PubMed: 21073339 21073339

[5] Tacke F, Weiskirchen R (2012) Update on hepatic stellate cells: pathogenic role in liver fibrosis and novel isolation techniques. Expert Rev Gastroenterol Hepatol 6: 67–80 10.1586/egh.11.92. PubMed: 22149583 22149583

[6] Friedman SL (1993) Seminars in medicine of the BethIsrael Hospital, Boston. The cellular basis of hepatic fibrosis. Mechanisms and treatment strategies. N Engl J Med 328: 1828–1835. PubMed: 8502273. 10.1056/NEJM199306243282508 8502273

[7] Friedman SL (2004) Stellate cells: a moving target in hepatic fibrogenesis. Hepatology 40: : 1041–1043. PubMed: 15486918. 10.1002/hep.20476 15486918

[8] Ikegami T, Zhang Y, Matsuzaki Y (2007) Liver fibrosis: possible involvement of EMT. Cells Tissues Organs 185: : 213–221. PubMed: 17587827. 10.1159/000101322 17587827

[9] Omenetti A, Bass LM, Anders RA, Clemente MG, Francis H, et al (2011) Hedgehog activity, epithelial-mesenchymal transitions, and biliary dysmorphogenesis in biliary atresia. Hepatology 53: 1246–1258 10.1002/hep.24156. PubMed: 21480329 21480329 PMC3074103

[10] Nitta T, Kim JS, Mohuczy D, Behrns KE (2008) Murine cirrhosis induces hepatocyte epithelial mesenchymal transition and alterations in survival signaling pathways. Hepatology 48: 909–919 10.1002/hep.22397. PubMed: 18712785 18712785 PMC4118693

[11] Zhu NL, Asahina K, Wang J, Ueno A, Lazaro R, et al (2012) Hepatic stellate cell-derived delta-like homolog 1 (DLK1) protein in liver regeneration. J Biol Chem 287: 10355–10367 10.1074/jbc.M111.312751. PubMed: 22298767 22298767 PMC3322997

[12] Zhu Q, Zou L, Jagavelu K, Simonetto DA, Huebert RC, et al (2012) Intestinal decontamination inhibits TLR4 dependent fibronectin-mediated cross-talk between stellate cells and endothelial cells in liver fibrosis in mice. J Hepatol 56: 893–899 10.1016/j.jhep.2011.11.013. PubMed: 22173161 22173161 PMC3307873

[13] Qiang H, Lin Y, Zhang X, Zeng X, Shi J, et al. (2006) Differential expression genes analyzed by cDNA array in the regulation of rat hepatic fibrogenesis. Liver Int 26: : 1126–1137. PubMed: 17032414. 10.1111/j.1478-3231.2006.01353.x 17032414

[14] Zhong W, Shen WF, Ning BF, Hu PF, Lin Y, et al (2009) Inhibition of extracellular signal-regulated kinase 1 by adenovirus mediated small interfering RNA attenuates hepatic fibrosis in rats. Hepatology 50: 1524–1536 10.1002/hep.23189. PubMed: 19787807 19787807

[15] Berasain C, Herrero JI, García-Trevijano ER, Avila MA, Esteban JI, et al (2003) Expression of Wilms' tumor suppressor in the liver with cirrhosis: relation to hepatocyte nuclear factor 4 and hepatocellular function. Hepatology 38: 148–157 10.1053/jhep.2003.50269. PubMed: 12829997 12829997

[16] Yue HY, Yin C, Hou JL, Zeng X, Chen YX, et al (2010) Hepatocyte nuclear factor 4alpha attenuates hepatic fibrosis in rats. Gut 59: 236–246 10.1136/gut.2008.174904. PubMed: 19671543 19671543

[17] Ambros V (2004) The functions of animal microRNAs. Nature 43: 350–355 10.1038/nature02871. PubMed: 15372042 15372042

[18] Png KJ, Halberg N, Yoshida M, Tavazoie SF (2011) A microRNA regulon that mediates endothelial recruitment and metastasis by cancer cells. Nature 481: 190–194 10.1038/nature10661. PubMed: 22170610 22170610

[19] Pedersen IM, Cheng G, Wieland S, Volinia S, Croce CM, et al. (2007) Interferon modulation of cellular microRNAs as an antiviral mechanism. Nature 449: : 919–922. PubMed: 17943132. 10.1038/nature06205 PMC2748825 17943132

[20] Thum T, Gross C, Fiedler J, Fischer T, Kissler S, et al (2008) MicroRNA-21 contributes to myocardial disease by stimulating MAP kinase signalling in fibroblasts. Nature 456: 980–984 10.1038/nature07511. PubMed: 19043405 19043405

[21] Wei J, Feng L, Li Z, Xu G, Fan X (2013) MicroRNA-21 activates hepatic stellate cells via PTEN/Akt signaling. Biomed Pharmacother 67: 387–392 10.1016/j.biopha.2013.03.014. PubMed: 23643356 23643356

[22] Marquez RT, Bandyopadhyay S, Wendlandt EB, Keck K, Hoffer BA, et al (2010) Correlation between microRNA expression levels and clinical parameters associated with chronic hepatitis C viral infection in humans. Lab Invest 90: 1727–1736 10.1038/labinvest.2010.126. PubMed: 20625373 20625373

[23] Liu LZ, Li C, Chen Q, Jing Y, Carpenter R, et al (2011) MiR-21 induced angiogenesis through AKT and ERK activation and HIF-1α expression. PLoS One 6: e19139 10.1371/journal.pone.0019139. PubMed: 21544242 21544242 PMC3081346

[24] Ling M, Li Y, Xu Y, Pang Y, Shen L, et al (2012) Regulation of miRNA-21 by reactive oxygen species-activated ERK/NF-κB in arsenite-induced cell transformation. Free Radic Biol Med 52: 1508–1518 10.1016/j.freeradbiomed.2012.02.020 PubMed: 22387281. 22387281

[25] Huang TH, Wu F, Loeb GB, Hsu R, Heidersbach A, et al (2009) Up-regulation of miR-21 by HER2/neu signaling promotes cell invasion. J Biol Chem 284: 18515–18524 10.1074/jbc.M109.006676. PubMed: 19419954 19419954 PMC2709372

[26] Vogel S, Piantedosi R, Frank J, Lalazar A, Rockey DC, et al.. (2000) An immortalized rat liver stellate cell line (HSC-T6): a new cell model for the study of retinoid metabolism in vitro. J Lipid Res 41: 882–893. PubMed: 10828080. 10828080

[27] Ma J, Li F, Liu L, Cui D, Wu X, et al (2009) Raf kinase inhibitor protein inhibits cell proliferation but promotes cell migration in rat hepatic stellate cells. Liver Int 29: 567–574 10.1111/j.1478-3231.2009.01981.x PubMed: 19323783 19323783

[28] Chen Y, Gorski DH (2008) Regulation of angiogenesis through a microRNA (miR-130a) that down-regulates antiangiogenic homeobox genes GAX and HOXA5. Blood 111: 1217–1226. PubMed: 17957028. 10.1182/blood-2007-07-104133 PMC2214763 17957028

[29] Wang T, Zhang L, Shi C, Sun H, Wang J, et al (2012) TGF-β-induced miR-21 negatively regulates the antiproliferative activity but has no effect on EMT of TGF-β in HaCaT cells. Int J Biochem Cell Biol 44: 366–376 10.1016/j.biocel.2011.11.012. PubMed: 22119803 22119803

[30] Noetel A, Kwiecinski M, Elfimova N, Huang J, Odenthal M (2012) microRNA are central players in anti- and profibrotic gene regulation during liver fibrosis. Front Physiol 3: 49 10.3389/fphys.2012.00049. PubMed: 22457651 22457651 PMC3307137

[31] Jiao J, Friedman SL, Aloman C (2009) Hepatic fibrosis. Curr Opin Gastroenterol 25: 223–229. PubMed: 19396960. 10.1097/mog.0b013e3283279668 PMC2883289 19396960

[32] Lee UE, Friedman SL (2011) Mechanisms of hepatic fibrogenesis. Best Pract Res Clin Gastroenterol 25: 195–206 10.1016/j.bpg.2011.02.005. PubMed: 21497738 21497738 PMC3079877

[33] Kwiecinski M, Elfimova N, Noetel A, Töx U, Steffen HM, et al (2012) Expression of platelet-derived growth factor-C and insulin-like growth factor I in hepatic stellate cells is inhibited by miR-29. Lab Invest 92: 978–987 10.1038/labinvest.2012.70. PubMed: 22565577 22565577

[34] Lakner AM, Steuerwald NM, Walling TL, Ghosh S, Li T, et al (2012) Inhibitory effects of microRNA 19b in hepatic stellate cell-mediated fibrogenesis. Hepatology 56: 300–310 10.1002/hep.25613. PubMed: 22278637 22278637 PMC3342471

[35] Pandit KV, Milosevic J, Kaminski N (2011) MicroRNAs in idiopathic pulmonary fibrosis. Transl Res 157: 191–199 10.1016/j.trsl.2011.01.012. PubMed: 21420029 21420029

[36] Niu J, Shi Y, Tan G, Yang CH, Fan M, et al (2012) DNA damage induces NF-κB-dependent microRNA-21 up-regulation and promotes breast cancer cell invasion. J Biol Chem 287: 21783–21795 10.1074/jbc.M112.355495. PubMed: 22547075 22547075 PMC3381141

[37] Bakirtzi K, Hatziapostolou M, Karagiannides I, Polytarchou C, Jaeger S, et al (2011) Neurotensin signaling activates microRNAs-21 and -155 and Akt, promotes tumor growth in mice, and is increased in human colon tumors. Gastroenterology 141: 1749–1761.e1 10.1053/j.gastro.2011.07.038. PubMed: 21806946 21806946 PMC4442678

[38] Chen YP, Jin X, Xiang Z, Chen SH, Li YM (2013) Circulating MicroRNAs as potential biomarkers for alcoholic steatohepatitis. Liver Int 33: 1257–1265 10.1111/liv.12196. PubMed: 23682678 23682678

[39] Murakami Y, Tanahashi T (2013) Analysis of circulating microRNA by microarray in liver disease. Methods Mol Biol 1024: 173–182 10.1007/978-1-62703-453-113. PubMed: 23719950 23719950

[40] Kostadinova R, Montagner A, Gouranton E, Fleury S, Guillou H, et al (2012) GW501516-activated PPARβ/δ promotes liver fibrosis via p38-JNK MAPK-induced hepatic stellate cell proliferation. Cell Biosci 2: 34 10.1186/2045-3701-2-34. PubMed: 23046570 23046570 PMC3519722

[41] Kuracha MR, Burgess D, Siefker E, Cooper JT, Licht JD, et al (2011) Spry1 and Spry2 are necessary for lens vesicle separation and corneal differentiation. Invest Ophthalmol Vis Sci 52: 6887–6897 10.1167/iovs.11-7531. PubMed: 21743007 21743007 PMC3176024

[42] Chow SY, Yu CY, Guy GR (2009) Sprouty2 interacts with protein kinase C delta and disrupts phosphorylation of protein kinase D1. J Biol Chem 284: 19623–19636 10.1074/jbc.M109.021600. PubMed: 19458088 19458088 PMC2740588

[43] Hacohen N, Kramer S, Sutherland D, Hiromi Y, Krasnow MA (1998) Sprouty encodes a novel antagonist of FGF signaling that patterns apical branchingof the Drosophila airways. Cell 92: 253–263. PubMed: 9458049. 10.1016/s0092-8674(00)80919-8 9458049

[44] Frey MR, Carraro G, Batra RK, Polk DB, Warburton D (2011) Sprouty keeps bowel kinases regular in colon cancer, while miR-21 targets Sprouty. Cancer Biol Ther 11: 122–124. PubMed: 21124074. 10.4161/cbt.11.1.14176 21124074

[45] Kwak HJ, Kim YJ, Chun KR, Woo YM, Park SJ, et al (2011) Downregulation of Spry2 by miR-21 triggers malignancy in human gliomas. Oncogene 30: 2433–2442 10.1038/onc.2010.620. PubMed: 21278789 21278789

[46] Xu WP, Yi M, Li QQ, Zhou WP, Cong WM, et al (2013) Perturbation of MicroRNA-370/Lin-28 homolog A/nuclear factor kappa B regulatory circuit contributes to the development of hepatocellular carcinoma. Hepatology 58: 1977–1991 10.1002/hep.26541. PubMed: 23728999 23728999

