# Supplementary figures and images for: MiR-21 Simultaneously Regulates ERK1 Signaling in HSC Activation and Hepatocyte EMT in Hepatic Fibrosis
Source: PLoS One. 2014 Oct 10;9(10):e108005. doi: 10.1371/journal.pone.0108005 (PMC4193742; doi:10.1371/journal.pone.0108005)

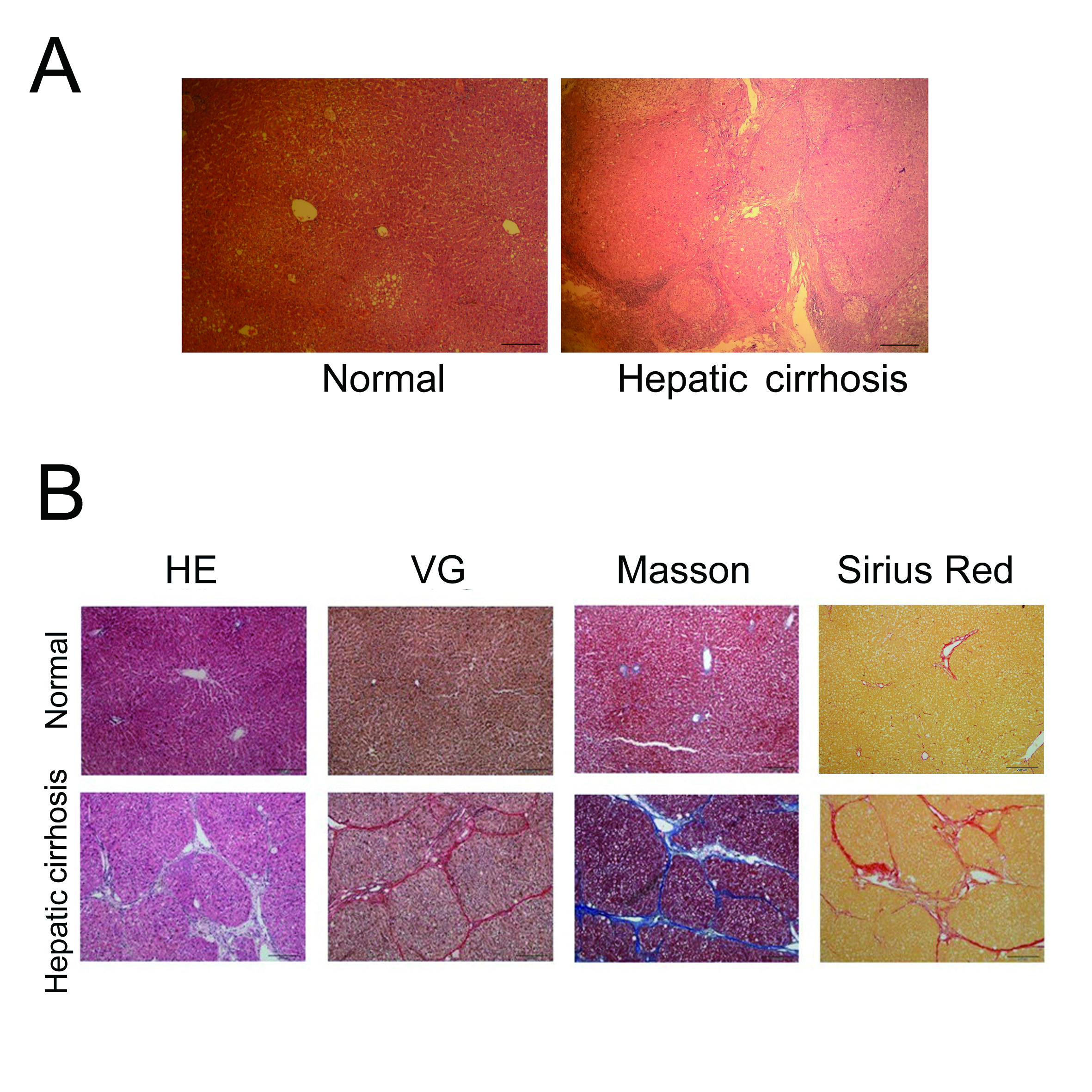

Supplement: Figure S1 — Histopathologic examination of the liver tissue of cirrhotic patients and rats. Histopathologic examination of the liver tissue from the patient with hepatic cirrhosis (A). H&E staining of the liver tissue from rats with dimethylnitrosamine-induced liver cirrhosis. The presence of cirrhosis is further shown by Van Gieson (VG), Masson and Sirius Red staining (B). (TIF) [file pone.0108005.s001.tif]

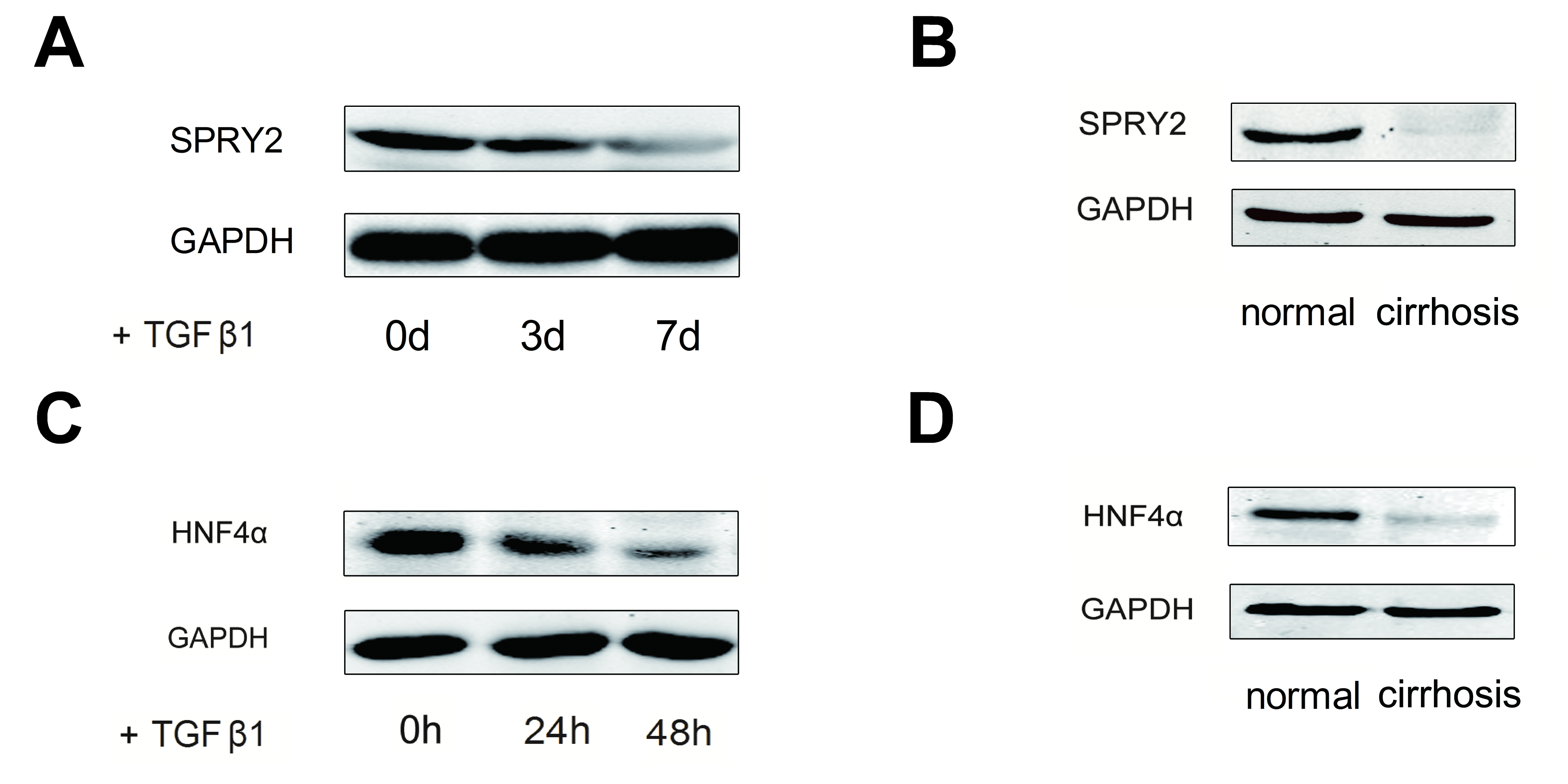

Supplement: Figure S2 — Protein levels of SPRY2 and HNF4α in TGFβ1 treated cells and cirrhotic rat liver tissues. The levels of SPRY2 and HNF4α were examined by Western blotting assays in primary HSCs treated with TGFβ1 for 7 days (A) and primary hepatocytes treated with TGFβ1 for 48 h (C). Representative images of levels of SPRY2 and HNF4α in cirrhotic rat liver tissue are shown (B and D). The proteins were extracted from normal (n = 3) and cirrhotic liver tissues (n = 3) randomly. The figures only showed the representative images. GAPDH was used as a loading control. (TIF) [file pone.0108005.s002.tif]

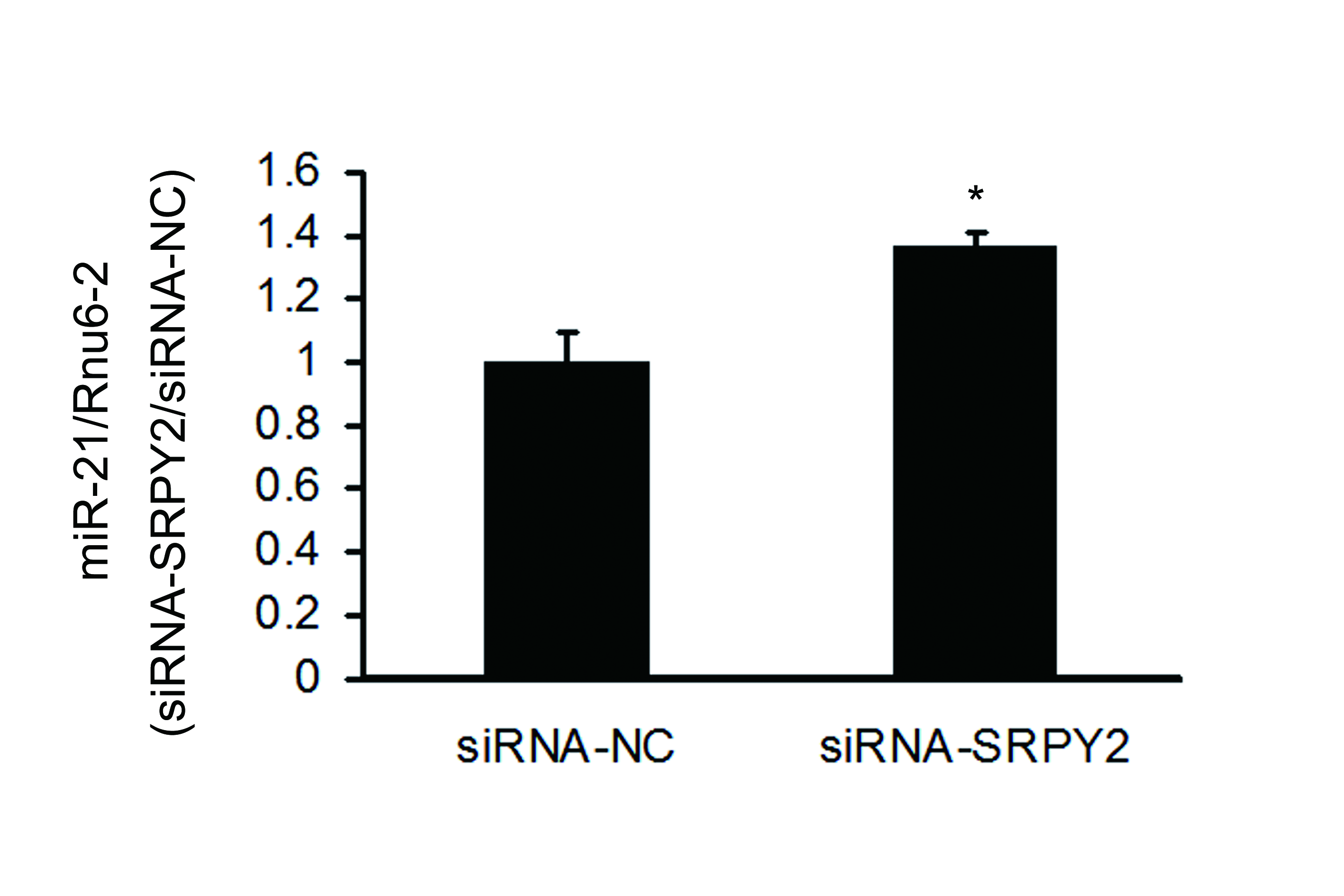

Supplement: Figure S3 — Effect of siRNA against SPRY2 on miR-21 level. Primary rat HSCs were transfected with siRNA against SPRY2. Cells were collected 48 h after siRNA delivery. Quantitative RT-PCR was carried out to detect miR-21 expression. Gene expression folds were normalized against the control. Each value represents the mean with the SD for triplicate samples. (*P<0.05). (TIF) [file pone.0108005.s003.tif]

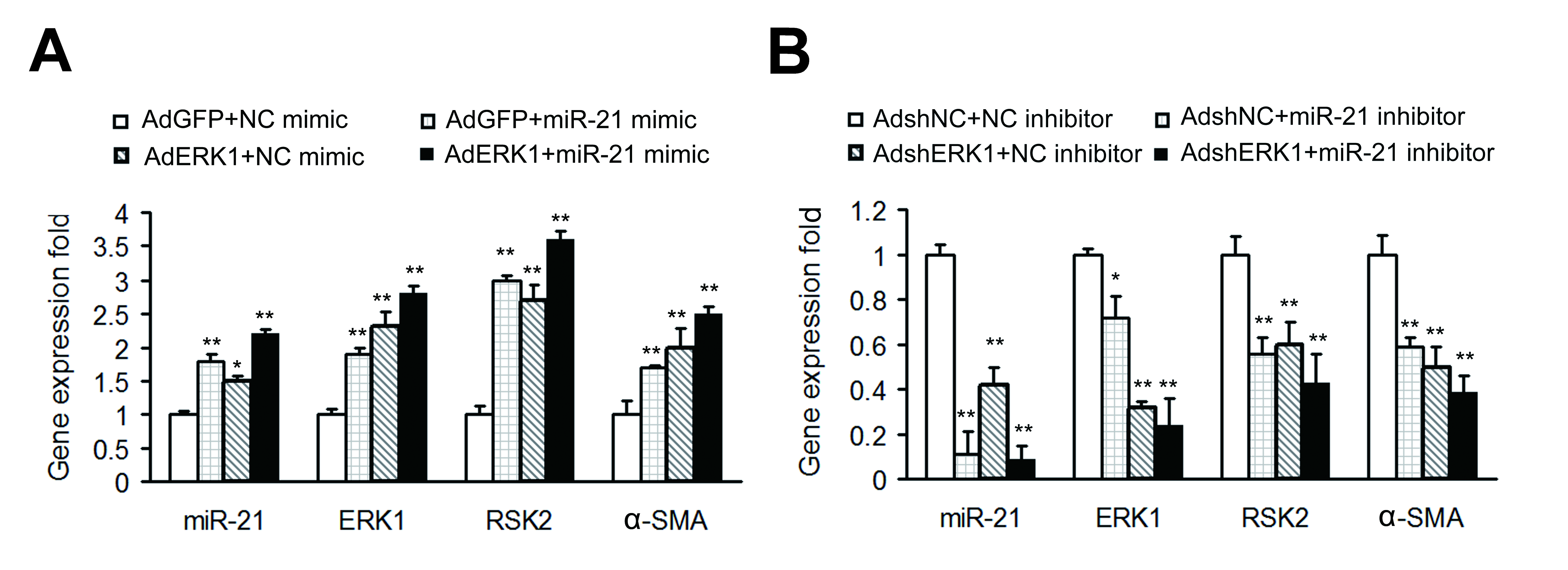

Supplement: Figure S4 — Associative action of ERK1 and miR-21. Primary rat HSCs were treated with AdERK1 and miR-21 mimic (A) or primary HSCs were treated with TGFβ1 for 48 h followed by AdshERK1 and miR-21 inhibitor transfection for 48 h (B). Quantitative RT-PCR was carried out to detect the levels of miR-21, ERK1, RSK2 and α-SMA. Gene expression folds were normalized against the control. Each value represents the mean with the SD for triplicate samples. (*P<0.05, **P<0.01). (TIF) [file pone.0108005.s004.tif]

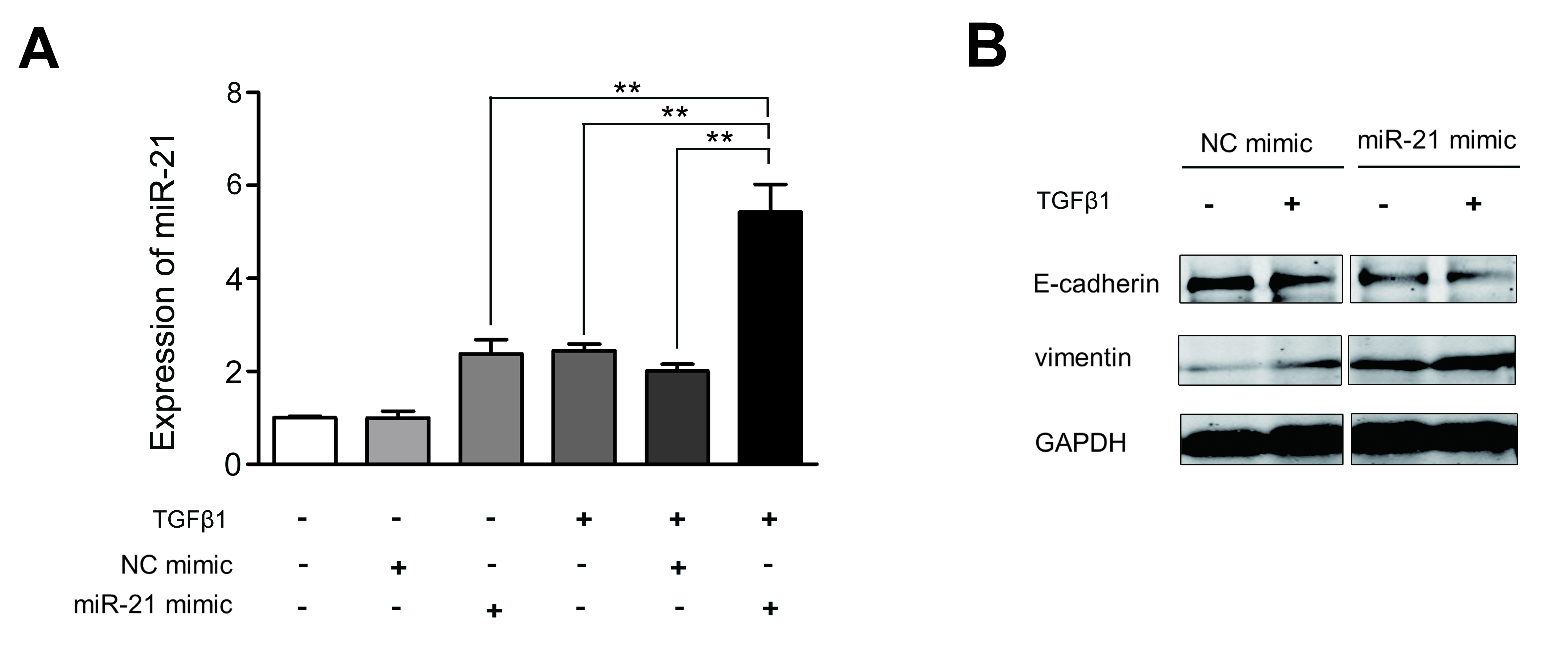

Supplement: Figure S5 — Combined effect of miR-21 mimics and TGFβ1 on EMT in primary rat hepatocytes. Primary rat hepatocytes were treated with miR-21 mimics and TGFβ1 for 48 h. MiR-21 level was examined by quantitative RT-PCR (A). Expression of E-cadherin and vimentin was examined by Western blotting assays (B). GADPH was used as a loading control. Blots representative of at least three independent experiments are shown. (TIF) [file pone.0108005.s005.tif]
