# Supplementary material for: MiR-21 Simultaneously Regulates ERK1 Signaling in HSC Activation and Hepatocyte EMT in Hepatic Fibrosis
Source: PLoS One. 2014 Oct 10;9(10):e108005. doi: 10.1371/journal.pone.0108005 (PMC4193742; doi:10.1371/journal.pone.0108005)
Supplement: Table S1 — Sequences for miRNA mimic and inhibitor, siRNA and 3′-UTR. (DOC) [file pone.0108005.s006.doc]

Supplementary table 1. Sequences for miRNA mimic and inhibitor, siRNA and 3′-UTR

| miR-21 mimic | Forward | 5′-UAGCUUAUCAGCUGAUGUUGA-3′ |
| --- | --- | --- |
|  | Reverse | 5′-AACAUCAGUCUGAUAAGCUAUU-3 |
| NC mimic | Forward | 5′-UUCUCCGAACGUGUCACGUTT-3′ |
|  | Reverse | 5′-ACGUGACACGUUCGGAGAATT-3′ |
| miR-21 inhibitor |  | 5′-UCAACAUCAGUCUGAUAAGCUA-3′ |
| NC inhibitor |  | 5′-CAGUACUUUUGUGUAGUAGUACAA-3′ |
| siRNA-ERK1 | Forward | 5′-CAUGGUCAG CUCAGCAUAUTT-3′ |
|  | Reverse | 5′-AUAUGCUGAGCUGACCAUGCC-3′ |
| siRNA- HNF4α | Forward | 5′-GCAGCCTACCCTCCATTAA-3′ |
|  | Reverse | 5′-TTAATGGAGGGTAGGCTGC-3′ |
| siRNA- SPRY2 | Forward | 5′-GCCAAGGGUUGCCUUAAAUTT-3′ |
|  | Reverse | 5′-AUUUAAGGCAACCCUUGGCTT-3′ |
| siRNA-NC | Forward | 5′-TTCTCCGAACGTGTCACG-3′ |
|  | Reverse | 5′-ACGTGACACGTTCGGAGAA-3′ |
| SPRY2 3′-UTR | Forward | 5′-CCGCTCGAGGGCACTGTTCA  TAGAGGGTTAG-3′ |
|  | Reverse | 5′-AAGGAAAAAAGCGGCCGGC  TTCCTGTAAGGCAAAATA-3′ |
| HNF4α 3′-UTR | Forward  Reverse | 5′-CCGCTCGAGGGTCACGGCAA  AGGAAGA-3′  5′-ATAAGAATGCGGCCGCTGGG  AGTGGGCAGGGTT-3′ |
