# Supplementary material for: MiR-21 Simultaneously Regulates ERK1 Signaling in HSC Activation and Hepatocyte EMT in Hepatic Fibrosis
Source: PLoS One. 2014 Oct 10;9(10):e108005. doi: 10.1371/journal.pone.0108005 (PMC4193742; doi:10.1371/journal.pone.0108005)
Supplement: Table S2 — Primer sequences for real-time polymerase chain reaction. (DOC) [file pone.0108005.s007.doc]

Supplementary table 2. Primer sequences for real-time polymerase chain reaction

| β-actin | Forward | 5'-ACCCACACTGTGCCCATCTATG-3' |
| --- | --- | --- |
|  | Reverse | 5'-AGAGTACTTGCGCTCAGGAGGA-3' |
| U6 | Forward | 5'-CTCGCTTCGGCAGCACA-3' |
|  | Reverse | 5'-AACGCTTCACGAATTTGCGT-3' |
| miR-21 | Forward | 5'-GCCCTAGCTTATCAGACTGA-3' |
|  | Reverse | 5'-GTGCAGGGTCCGAGGT-3' |
| SPRY2 | Forward | 5′-ATCAGAGCCATCCGAAACAC-3′ |
|  | Reverse | 5′-CCTTGTACTGCTCCGAGACC-3′ |
| HNF4α | Forward | 5′-AAATGTGCAGGTGTTGACCA-3′ |
|  | Reverse | 5′-CACGCTCCTCCTGAAGAATC-3′ |
| α-SMA | Forward | 5'-CCGAGATCTCACCGACTACC -3' |
|  | Reverse | 5'-TCCAGAGCGACATAGCACAG -3' |
| TGFβ1 | Forward | 5'- GACCGCAACAACGCAATCTA -3' |
|  | Reverse | 5'- ACCAAGGTAACGCCAGGAAT -3' |
| TIMP1 | Forward | 5′-TCCCCAGAAATCATCGAGAC-3′ |
|  | Reverse | 5′-TCAGATTATGCCAGGGAACC-3′ |
| MMP2 | Forward | 5′-CGGTTTATTTGGCGGACAGT-3′ |
|  | Reverse | 5′- TGGCTTGGGGTATCCTCTTTC-3′ |
| MMP9 | Forward | 5′-CCACCGAGCTATCCACTCAT-3′ |
|  | Reverse | 5′- GTCCGGTTTCAGCATGTTTT-3′ |
| MMP13 | Forward | 5′-AGGCCTTCAGAAAAGCCTTC-3′ |
|  | Reverse | 5′- GAGCTGCTTGTCCAGGTTTC-3′ |
| Collagen type І | Forward | 5′-CCGTGACCTCAAGATGTGCC-3′ |
|  | Reverse | 5′- GCTCATACCTTCGCTTCCAA-3′ |
| Collagen type Ⅲ | Forward | 5′-AGGCCAATGGCAATGTAAAG-3′ |
|  | Reverse | 5′- TATTGGTGGGTGAAACAGCA-3′ |
| E-cadherin | Forward | 5′-GGGTTGTCTCAGCCAATGTT-3′ |
|  | Reverse | 5′-CACCAACACACCCAGCATAG-3′ |
| vimentin | Forward | 5′-AGATCGATGTGGACGTTTCC-3′ |
|  | Reverse | 5′-CACCTGTCTCCGGTATTCGT-3′ |
| slug | Forward | 5′- AGAAGCCCAACTACAGCGAACT-3′ |
|  | Reverse | 5′- CAGAGGGGAGTGGAATGGAAC-3′ |
| ERK1 | Forward | 5′-TCCAAGGGCTACACCAAATC-3′ |
|  | Reverse | 5′-AGGTAGTTTCGGGCCTTCAT-3′ |
| RSK2 | Forward | 5′-CTGACTGGTGGTCCTTTGGT-3′ |
|  | Reverse | 5′-GACAAAGAACGGGTGACGTT-3′ |
| ALB | Forward  Reverse | 5′-TGCAGGCTTGCTGTGATAAG-3′  5′-AGTAATCGGGGTGCCTTCTT-3′ |
| CYP1α2 | Forward | 5′-CAGGTTCCCAAAGGTCTGAA-3′ |
|  | Reverse | 5′-TTCACTAGGGCCTGCTTGAT-3′ |
| AFP | Forward | 5′-TACGTCCCTCCACCATTCTC-3′ |
|  | Reverse | 5′-ATCCTGGTCTTTGCAGCACT-3′ |
