# Supplementary material for: MiR-21 Simultaneously Regulates ERK1 Signaling in HSC Activation and Hepatocyte EMT in Hepatic Fibrosis
Source: PLoS One. 2014 Oct 10;9(10):e108005. doi: 10.1371/journal.pone.0108005 (PMC4193742; doi:10.1371/journal.pone.0108005)
Supplement: Table S3 — Primary antibodies used for western blotting analysis. (DOC) [file pone.0108005.s008.doc]

Supplementary table 3. Primary antibodies used for western blot analysis

| Antigens | species | Poly/monoclonal | Manufacturer | Dilution |
| --- | --- | --- | --- | --- |
| SPRY2 | mouse | polyclonal | Santa Crutz Biotechnology, Inc. | 1:800 |
| HNF4α | mouse | monoclonal | R&D Systems, Inc. | 1:500 |
| ERK 1/2 | rabbit | polyclonal | Cell Signaling Technology, Inc. | 1:1000 |
| p-ERK 1/2 | rabbit | monoclonal | Cell Signaling Technology, Inc. | 1:1000 |
| RAS | rabbit | monoclonal | Cell Signaling Technology, Inc. | 1:1000 |
| RSK2 | rabbit | monoclonal | Santa Crutz Biotechnology, Inc. | 1:200 |
| E-cadherin | mouse | polyclonal | Becton Dickinson and Company. | 1:3000 |
| vimentin | mouse | monoclonal | Dako Company. | 1:1000 |
| ALB | rabbit | polyclonal | Santa Crutz Biotechnology, Inc. | 1:1000 |
| MMP2 | rabbit | polyclonal | Boster Biological Technology. | 1:200 |
| TIMP1 | rabbit | polyclonal | Boster Biological Technology. | 1:500 |
| α-SMA | rabbit | monoclonal | Boster Biological Technology. | 1:200 |
| Collagen typeⅠ | rabbit | polyclonal | Boster Biological Technology. | 1:200 |
| GAPDH | mouse | polyclonal | Bioworld Technology, Inc. | 1:3000 |
